# A Medication Management App (Smart-Meds) for Patients After an Acute Coronary Syndrome: Pilot Pre-Post Mixed Methods Study

**DOI:** 10.2196/50693

**Published:** 2025-01-23

**Authors:** Frederic Ehrler, Liliane Gschwind, Hamdi Hagberg, Philippe Meyer, Katherine Blondon

**Affiliations:** 1 Information Systems Directorate, University Hospital of Geneva, Geneva, Switzerland; 2 Department of Pharmacy, University Hospital of Geneva, Geneva, Switzerland; 3 Service of Cardiology, University Hospital of Geneva, Geneva, Switzerland; 4 Medicine Faculty, University of Geneva, Geneva, Switzerland; 5 Medical Directorate, University Hospital of Geneva, Geneva, Switzerland

**Keywords:** medication adherence, gamified app, narration, acute coronary syndrome, beliefs about medication, self-reported adherence, pilot study, usability evaluation, storytelling component

## Abstract

**Background:**

Medication nonadherence remains a significant challenge in the management of chronic conditions, often leading to suboptimal treatment outcomes and increased health care costs. Innovative interventions that address the underlying factors contributing to nonadherence are needed. Gamified mobile apps have shown promise in promoting behavior change and engagement.

**Objective:**

This pilot study aimed to evaluate the efficacy and usability of a gamified mobile app that used a narrative storytelling approach to enhance medication adherence among patients following acute coronary syndrome (ACS). The study aimed to assess changes in participants’ beliefs about medication and self-reported adherence before and after the intervention. Additionally, user feedback regarding the narrative component of the app was gathered.

**Methods:**

Overall, 18 patients who recently experienced ACS were recruited for a 1-month intervention using the gamified app. Participants’ beliefs about medication and self-reported adherence were assessed using standardized scales pre- and postintervention. The app’s usability was also evaluated through a postintervention questionnaire. Statistical analyses were performed to determine the significance of changes in belief and adherence scores.

**Results:**

Although 33% (6/18) of the participants did not use the intervention more than once, the remaining 12 remained engaged during the 30 days of the study. The results did not indicate a significant improvement in participants’ beliefs about medication following the intervention. However, self-reported adherence significantly improved (*P*<.05) after the intervention with a mean score going from 29.1 (SD 6.9) to 32.4 (SD 5.6), with participants demonstrating a greater self-efficacy to their prescribed medication regimen. However, the results did not indicate a significant improvement in participants’ beliefs about medication. With a mean average score of 80.6, the usability evaluation indicates a good usability rating for the gamified app. However, the narrative storytelling component of the app was not favored by the participants, as indicated by their feedback.

**Conclusions:**

This pilot study suggests that a gamified mobile app using narration may effectively enhance medication self-efficacy and positively influence patients’ beliefs about medication following ACS. However, the narrative component of the app did not receive favorable feedback from participants. Future research should focus on exploring alternative methods to engage participants in the app’s narrative elements while maintaining the positive impact on adherence and beliefs about medication observed in this study.

## Introduction

Medication nonadherence is a well-identified health care issue, particularly for chronic diseases. Poor adherence worsens clinical outcomes and induces higher downstream rehospitalization rates as well as a higher use of resources [1]. Despite the physicians’ efforts to convey the importance of the medications they prescribe, patients still find several intentional or unintentional reasons for deviating from their treatment plan [2]. Prior research reports that the most common factors associated with nonadherence are forgetfulness (50%), having other medications to take (20%), and being symptom-free (20%) [3]. The risk of poor adhesion is further increased with the medication regimen complexity, which increases with each decision about taking medication that a patient needs to make [4].

After an acute coronary syndrome (ACS), secondary cardiovascular prevention recommendations mainly involve lifestyle changes (eg, physical activity, smoking, or diet) and adherence to the prescribed drug regimen [5]. Patients with ACS are at particular risk of failing to adhere to their medication regimen since they may lack comprehension of medication importance, and have difficulty accessing medication, or affording the medication [6]. Additionally, medications used to treat ACS can have significant side effects that can make it difficult to take them regularly [7]. Patients with ACS may also need to take multiple medications, and there is a risk of drug interactions between them [8]. Moreover, the various medications used to treat ACS require regular monitoring to ensure they are working properly and to monitor the side effects. Finally, the medications used to treat ACS often require a longer time, which can be difficult for some patients to adhere to [9].

Mobile health apps provide new opportunities to support medication adherence [10]. First, they can remind users to take their medication on time. This can help ensure that users do not forget to take their medication or take incorrect doses. For instance, a meta-analysis of SMS text messaging interventions to improve adherence to medication in chronic diseases showed that SMS text message reminders were associated with increased odds of being adherent [11]. Second, mobile apps can track patients’ medication use and provide feedback on their progress. They can offer personalized advice for treatment and behavioral change support, as well as facilitate communication between patients and their health care professionals [12]. This can help patients keep track of their medication use and identify any issues that may be preventing them from taking their medication as prescribed. Finally, mobile apps can connect users with health care professionals and support groups to provide additional motivation and help. This can help patients stay on track with their medication use and provide emotional support when needed.

Gamification for health behavior change involves applying game design elements and principles to encourage and motivate individuals to adopt healthier behaviors. It leverages techniques such as rewards, challenges, competition, and progress tracking to engage users in activities that promote better health outcomes. Examples include fitness apps that award points for completing workouts, digital platforms that encourage healthy eating through virtual rewards, and wearable devices that gamify physical activity by setting goals and providing feedback. By making health-related tasks more enjoyable and interactive, gamification aims to increase user motivation, adherence to health goals, and overall well-being [13]. Gamification is a mechanism that has proven to be efficient in promoting behavior change [14]. Yet it has not been largely assessed in the context of medication adherence. Moreover, to our knowledge, there are currently no apps with gamification that target the Swiss market with the available medications in this country [15,16].

In an attempt to boost adherence, a multidisciplinary team of health professionals, informaticians, and patients in a cardiac rehabilitation (CR) program worked together to develop an innovative app with gamification strategies named “Smart-Meds.”

The main objective of this study was to evaluate the adoption, usability, and satisfaction of Smart-Meds among users enrolled in an outpatient CR program. We also explored the impact of app use on medication adherence and beliefs.

## Methods

### Study Design

This is a pilot pre-post study aimed at assessing the impact on participants’ self-efficacy regarding their medication regimens and their beliefs about medication efficacy following the use of the Smart-Meds app for 1 month.

### Primary and Secondary Outcome

The primary outcome is the Self-Efficacy for Appropriate Medication Use Scale (SEAMS), and the secondary outcomes are the Beliefs About Medication Questionnaire (BMQ) and the System Usability Scale (SUS).

### Participants

We included adults (>18 years) who were treated for an ACS in the past month and who owned an Android or iPhone. We excluded participants who did not speak conversational French.

### Sample Size

In this pilot pre-post study, the sample size was determined using the rule of thumb for pilot studies, which suggests a minimum of 12 participants per group to provide an initial estimate of effect sizes and variability [17]. This sample size is considered adequate for assessing feasibility and refining study protocols, while not intended for definitive hypothesis testing. The selected sample size allows for the identification of trends and potential issues that may inform the design of a subsequent, fully powered study.

### Recruitment

We enrolled voluntary participants entering a CR program at the University Hospital of Geneva. Patients were recruited during round table sessions by an investigator presenting the study. After providing their consent, the participants received help if needed to install and use the app on their smartphones.

### Ethical Consideration

An ethical application was made to the hospital’s ethical committee. The ethics committee considered that this research was targeted mainly to evaluate the application itself and could be considered as quality-related research. Therefore, they exempted us from ethical approval. Informed consent was signed by all participants prior to the inclusion in the study. All data collected in the study have been anonymized by using unique identifiers before analysis, ensuring that no personal information could be traced back to any individual. There was no need for compensation, and no images of individual participants were included in this paper and supplementary materials.

### Intervention

Smart-Meds is an app created following a participatory design. Users were involved all along its development, providing feedback at each step of the iterative cycles of formative evaluation [18]. The users participating in the app conception were patients participating in or having recently completed the 6-week CR program. The app’s main aim is to empower users to manage their medications, using gamification strategies to motivate users to report their intakes. The app allows users to easily enter medications into their personal medication plan through barcode scanning of the drug boxes. Besides avoiding transcription errors, this process ensures that the correct medication is entered (pharmacies may provide different generics of a drug), and the user only has the dosage and schedule to enter. Users can set reminders about when to take their medications and have links to the Swiss patient information web page about their drugs. For the standard cardiovascular drugs, our team also developed simplified information content about indications and side effects that were adapted to low health literacy levels. We also created an educational section in the app about coronary heart disease, based on the CR program materials.

To increase users’ motivation to report their medication intake, we relied on gamification mechanisms. The core mechanism is a narration whose daily stages of a motivational story are unlocked by reporting medication intake. Narrative has been demonstrated to be a relevant mechanism that can foster behavior change [19]. Narratives can help bridge the gap between intention and action. The health action process approach suggests that people may not act on a desired behavior for different reasons: those who are not (yet) motivated to do so are nonintenders, while intenders may be motivated but unable to put their intention into action [20]. According to this approach, planning strategies are essential in aiding intenders to close this gap. These strategies involve specifying when, where, and how to carry out the desired behavior (action planning) and anticipating potential obstacles and preparing ways to overcome them (coping planning). Narratives are particularly useful in this regard; they focus on specific characters, their actions and motivations, and present events in a temporal and causal structure. Therefore, characters can act as role models, demonstrating how to turn intention into action, what to expect in terms of challenges, and how to navigate them successfully [21].

This story was designed to increase engagement and reinforce the concepts of the “health action process approach” model [22] and is inspired by an annual outing for patients with ACS at the Cardiac Rehabilitation Center of the University Hospital of Geneva. The story consists of 30 episodes. The average textual length of each episode is 470 characters.

**Figure 1.**
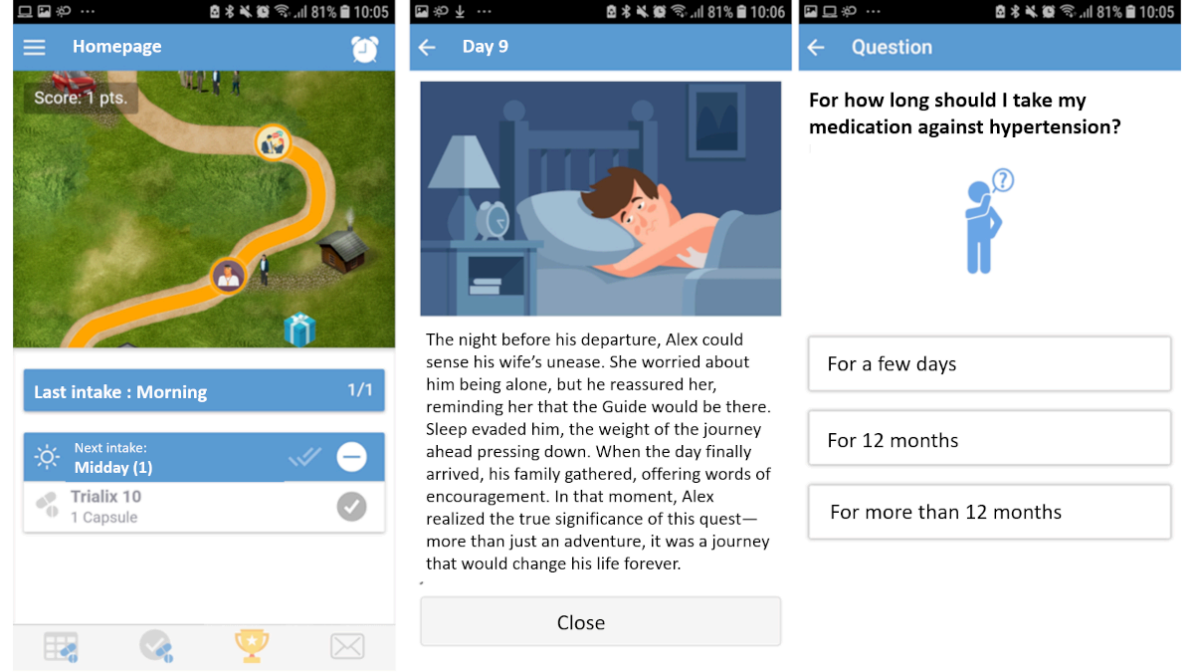
Screenshot of the app: the first screen displays the story stages unlocked by reporting medication, the second screen displays a part of the story, and the third screen displays an example of the quiz (translated into English from the original French version).

Another gamified mechanism implemented in the app is the progression since the user sees its progression toward storing through a visual path on the app (Figure 1).

Users can also test their knowledge about coronary heart disease and its management through daily quizzes. Finally, the app allows users to evaluate their cardiovascular risk factors to guide their lifestyle changes. A more detailed description of the app and its underlying framework is reported elsewhere [23].

### Study

#### Measures and Data Collection

Once recruited, participants completed questionnaires on demographic data and on medication adherence and beliefs (SEAMS and BMQ) [24,25]. SEAMS is a self-reported questionnaire with 13 items about how to manage one’s drugs in various situations (eg, change in routine, suspected side effects, and new prescriptions). The BMQ has 18 items, with subsets of questions on the nature of medication, their use by doctors, one’s personal need for a drug, and concerns about side effects. The participant then received the mobile app and received some help if necessary to install the app on their smartphones. The investigators also helped the participants to enter their treatment into the app. The participants were then instructed to use the app for 4 weeks at home without any interactions with the investigators or any recall.

After 4 weeks, in addition to the completion of a second SEAMS and BMQ, participants scored the app with the SUS. An investigator also conducted a semistructured oral interview in person or by phone. Nine open-ended questions were designed by the investigators based on a combination of deductive and inductive approaches. The investigation team started with the research objectives (deductive) and refined and expanded questions based on insights gained from initial data analysis and literature review (inductive). The selected questions explored reasons for satisfaction and app use and enquired about suggestions for improvements. The investigator audio-recorded the interviews or took session notes for a subsequent analysis. We also collected data about app use from the app logs (number of sessions, duration of session). Due to technical limitations, the log data were only captured when the participant was online at the time of app use. Only log sessions lasting more than 1 second were considered significant for this study.

#### Data Analysis

We report descriptive statistics of the demographic data to characterize our sample and of the use logs. We used a qualitative approach for the interviews, extracting common themes through iterative coding and comparisons of the data. SEAMS and BMQ scores are reported before and after the intervention and their distribution is compared using a chi-square analysis. Analyses were done using Microsoft Excel version 1808.

The study was carried out in French: as there was no validated translation available at the time of the study for the SEAMS, we proceeded with a translation or back-translation with 2 external consultants.

## Results

### Demographics

We recruited participants between February and April 2020. We report the results of the 18 participants who completed the study in Table 1 (of 37 participants screened for eligibility, 19 declined). Overall, participants were mainly male and Caucasian, with high socioeconomic status, which is representative of our targeted population. All participants had 4G connectivity. At the beginning of the study, half the participants monitored their blood pressure and physical activity.

**Table 1.** Participant characteristics (n=18).

Variable		Values
Week of program at enrollment (total of 6 weeks), mean (IQR)		2 (1-2.75)
Medications, mean (IQR)		5 (4.25-7.75)
**Age category (years), n (%)**		
	35-44	2 (11)
	45-54	5 (28)
	55-64	8 (44)
	65-74	3 (17)
**Sex, n (%)**		
	Male	16 (89)
	Female	2 (11)
**Educational attainment, n (%)**		
	High school	7 (39)
	College or higher	11 (61)
**Origin, n (%)**		
	Caucasian	14 (78)
	Other	4 (22)
**Private health insurance, n (%)**		
	Yes	12 (67)
	No	6 (33)
**Type of smartphone, n (%)**		
	Android	7 (39)
	iPhone	11 (61)
**Use of apps for health, n (%)**		
	Wellness	2 (11)
	Medical	6 (33)
	None	10 (56)
**Current monitored parameter, n (%)**		
	Blood pressure	10 (56)
	Weight	7 (39)
	Physical activity	9 (50)
	Diet	6 (33)
	Blood glucose	2 (11)

### Usage Pattern

All 18 participants installed and used Smart-Meds successfully. We see in Figure 1 that although every participant installed the app on the first day, we had an immediate dropout of one-third of the users. After that, the use remains stable until day 25.

On average, active participants used the app 3.76 (SD 1.28) sessions per day with a total of 64.39 (SD 21.55) seconds per day (Table 2). The highest app use was on the first day with an average of 4.67 sessions per participant of 2.5 minutes duration. App use drops rapidly after the first couple of days and persists at about 1x/day until the end of the 30 days.

**Table 2.** Use of the Smart-Meds app over the 30 days.

Day of the study	Daily user, n	Still active participants, n (%)	Sessions per active user, mean (SD)	App use duration per user (second), mean (SD)
1	18	18 (100)	4.67 (5.69)	147.98 (267.91)
2	11	13 (72)	6.29 (5.55)	89.31 (142.29)
3	8	12 (67)	3.13 (2.10)	60.68 (90.08)
4	9	12 (67)	3.89 (2.71)	67.96 (98.49)
5	9	12 (67)	4.71 (3.77)	74.88 (130.38)
6	9	12 (67)	3.00 (2.00)	85.26 (108.58)
7	6	12 (67)	4.89 (3.98)	56.02 (55.53)
8	9	12 (67)	3.22 (2.33)	57.05 (52.51)
9	8	12 (67)	2.29 (2.63)	40.92 (44.42)
10	8	12 (67)	4.00 (3.34)	80.94 (84.45)
11	6	12 (67)	3.00 (1.79)	68.02 (61.53)
12	9	12 (67)	4.57 (1.72)	42.53 (39.62)
13	9	12 (67)	5.50 (6.87)	54.11 (60.09)
14	8	12 (67)	5.43 (6.24)	43.88 (44.30)
15	9	12 (67)	4.43 (3.95)	48.54 (60.97)
16	9	12 (67)	2.20 (1.99)	66.43 (80.70)
17	8	12 (67)	3.00 (2.00)	72.16 (62.64)
18	8	12 (67)	6.88 (7.49)	48.56 (42.78)
19	8	12 (67)	3.71 (1.80)	73.35 (96.89)
20	9	12 (67)	4.50 (4.47)	75.48 (110.52)
21	9	12 (67)	2.00 (1.41)	41.47 (33.05)
22	6	12 (67)	2.33 (1.53)	77.99 (55.05)
23	8	12 (67)	2.20 (1.64)	48.77 (35.50)
24	7	12 (67)	4.00 (3.70)	60.96 (65.65)
25	9	12 (67)	3.50 (2.26)	38.39 (50.23)
26	10	12 (67)	2.11 (1.17)	77.14 (85.99)
27	10	11 (61)	2.43 (1.40)	52.93 (43.41)
28	8	9 (50)	4.63 (3.66)	76.94 (163.36)
29	8	9 (50)	3.71 (2.63)	43.39 (48.26)
30	7	7 (39)	2.67 (1.86)	59.55 (47.32)

### Pre-Post Evaluation of SEAMS and BMQ

**Figure 2.**
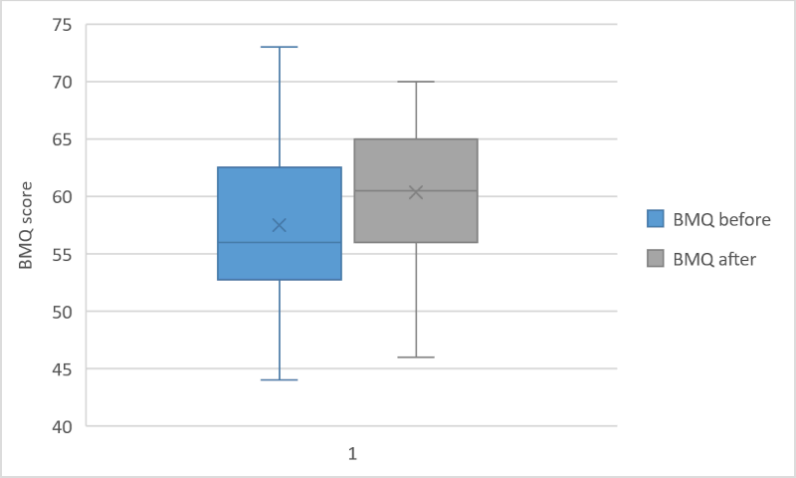
Boxplot of the BMQ score before and after the intervention period for the 18 participants. BMQ: Beliefs About Medication Questionnaire.

Although we did not find a significant change in the assessments of medical beliefs (BMQ, *P*=.09), the self-reported medication adherence score was significantly higher after 4 weeks (SEAMS, *P*=.02). Distribution of the SEAMS and BMQ scores can be visualized in Figures2  3.

**Figure 3.**
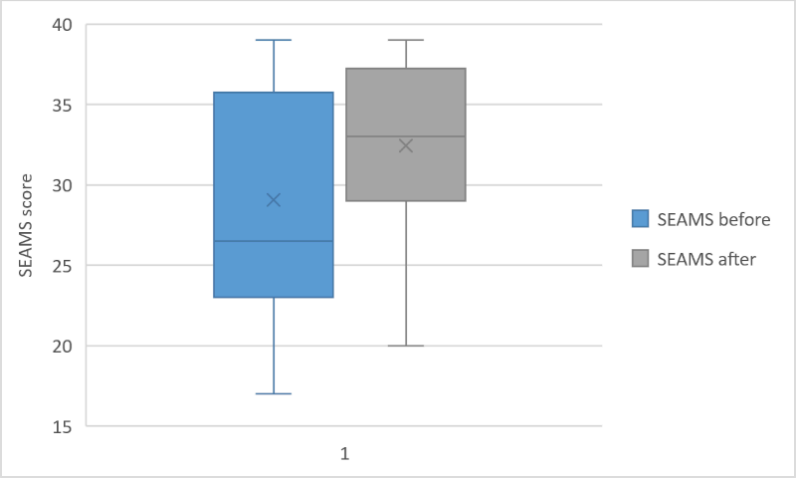
Boxplot of the SEAMS score before and after the intervention period for the 18 participants. SEAMS: Self-Efficacy for Appropriate Medication Use Scale.

### Semistructured Interview

In the semistructured interview, the 18 participants were overall very positive about the app, particularly when starting a new medication. Of the 18 participants, 5 (28%) liked being able to track their medication intake. One participant explained: “It’s very useful, because sometimes you can’t remember if you’ve taken the medication or not. With the app, I can validate taking the medication, and I do it as first action in the morning.” They were satisfied with the drug information and liked having an overview of all their medications, which they could share with their primary care physician. They appreciated its ease of use and found the barcode scanning an easy and fun way to enter their medications in the app. Despite some bugs linked to the modification of the recall time in the reminder functionalities during the study, the users thought having reminders was useful. They also found having pictures of their medications useful, especially with new drugs.

Of the 18 participants, 17 (94%) tested the quizzes and 15 (83%) enjoyed challenging their knowledge about their disease and their medications in this manner. In fact, 1 participant even suggested adding a reminder to take the quiz. Opinions about the motivational story were more varied because many participants did not engage with the story. Of the 18 participants, only 4 participants read the story until the end, and 1 participant suggested making it more interactive, where user choices affect the storyline. Half of the participants (9/18, 50%) reported the story as one of the less useful aspects of the app for them.

The participants did recognize that having a medication app was mainly useful early in the self-management process. Once they got into a routine to take the medication, the reminders were not as useful. In fact, 1 participant explained that taking his medications regularly was easy, but remembering to use the app was more difficult for him!

### System Usability Scale

**Figure 4.**
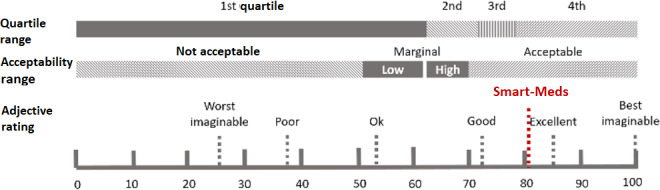
Results of the System Usability Scale (SUS) for the 18 participants.

Overall, the app was rated with a mean average score of 80.6 (SD 14.5), which may be interpreted as a good score according to Bangor et al [26]. The app was perceived between good and excellent (Figure 4).

## Discussion

### Principal Findings

Our pilot study revealed that participant satisfaction among users was high and that they would recommend the app to others. Our results show an improvement in the self-reported medication adherence scale after 4 weeks of app use. Even though gamification has been demonstrated successful in boosting behavior change in several contexts, it seems to have a limited impact on our specific population.

### Comparison to Prior Work

Although several recent studies have suggested that gamification can drive health behavior change, the type of gamification technique needs to be considered [27,28]. For our participants, the impact of the motivational story was very different from the quiz. Storytelling was considered as a game, whereas the quiz was more a verification of acquired knowledge, something that they valued.

The story was created with ups and downs to represent daily variations when coping with a challenge. We kept the story sequences short and used many illustrations to draw the reader’s attention. The users in our study did not demonstrate a strong interest in the motivational story. A plausible explanation is that the patients in our study were currently being treated for ACS, diagnosed in the past month [6]. We can suppose these participants were concerned about their current situation and did not find any added value from storytelling since their intrinsic motivation was already high [29,30].

The narrative approach has been used in other research. An article by Day [31] describes how storytelling has the potential to promote health literacy in patients. In the cardiology domain, Li et al [32] displayed an interactive video that depicted a model patient enacting a scenario with the patient experiencing acute myocardial infarction symptoms and going through the perceptual cognitive processes in decision-making. The psychoeducational intervention group reported greater positive changes than the control group in their attitudes.

The use of a quiz, however, another gamification technique, was well appreciated by the participants. Throughout the CR program, there are group discussions about heart disease, medications and side effects, and a healthy diet. They liked the idea of “checking” what knowledge they had acquired during the program. In fact, the quizzes were a way to monitor what they had understood and learned, rather than an outcome with the quiz score. Therefore, the participants had a much bigger interest in the quiz.

### Dropout

We observe in the log that one-third of the participants did only use the app once at the installation. This information does not correspond to the feedback of the patient during the semistructured interview. Indeed, during the interview, 14 patients reported using the app at least once per day, 3 patients twice per day, and 1 patient once every 2 days. The difference between the measured use and the reported one can have two reasons. First, research in various settings has demonstrated a difference between reported adherence and measured one [33]. The second reason is technical. Since the measure of adherence is recorded on the backend, if the patient is not connected to the internet when reporting his or her intake, that information is not logged.

### Adherence

We observe that self-reported adherence to medication improved over time. Prior studies have shown that a good understanding of one’s medication (why it is needed, how to take it, and potential side effects) is a driver for adherence [9,34]. Reading the simplified information facts in the app or self-testing with the quiz could have helped gain or maintain knowledge about medication during the study. Interestingly, the participants reported that the tracking functions were often not needed at this stage of their disease management: either they had already established a routine that suited them, or else they sometimes were low-tech and did not consider logging into the app regularly to track their medication intake [35,36]. Several participants considered this tracking as an additional, tedious task and therefore did not find tracking or reminders useful. The reminders were considered more useful when their routine was disrupted: this is commonly found in studies about adherence [37]. At this stage of the disease (CR program or right after the program), participants are still on sick leave at home, without the unexpected events that may occur from work-related tasks or travel issues.

### Other Contextual Elements

Participants enrolled in our study were from the CR program, with social support between peers, group sessions with health professionals, and daily physical activities in groups. In fact, patients often join a WhatsApp group to communicate with peers. This suggests other approaches to explore to help drive behavior changes, especially when the CR program ends, and “real life” begins again with work.

### Limitations

The first limitation of our study concerns the absence of a control group preventing to establish causality definitively. Without a control group for comparison, it becomes challenging to discern whether the observed changes in adherence behaviors and beliefs are solely attributable to the intervention or if they could be influenced by external factors or natural fluctuations over time. Additionally, the absence of a control group limits the researchers’ ability to account for potential confounding variables that may impact the outcomes of interest. Therefore, while the pre-post pilot study design provides valuable insights into the potential effects of the intervention, its findings must be interpreted cautiously, and further research using a controlled study design is warranted to confirm and generalize the observed results.

The second limitation of this pre-post scientific pilot study is the small sample size, which may render the study underpowered. With a limited number of participants, the study’s ability to detect significant changes in adherence behaviors and beliefs may be compromised. Small sample sizes can increase the likelihood of type II errors, where the study fails to detect real effects due to insufficient statistical power. Additionally, the generalizability of findings from a small sample size may be limited, as the characteristics and responses of a small group may not be representative of the broader population. Consequently, a cautious interpretation of the results is necessary, recognizing the potential limitations imposed by the small sample size on the study’s reliability and generalizability. Future research with larger sample sizes would be beneficial to confirm and extend the findings of this pilot study.

Third, we faced limitations to record app use when offline. This may have led to a bias in the reporting of the results, as several users were voluntarily disconnecting their smartphones from wireless networks to minimize connection costs. Therefore, we can expect that users were using the app more frequently than reported.

### Future Direction

Building on the findings of this pilot study, future research could explore more tailored storytelling approaches to enhance patient engagement and adherence to medication. Identifying narratives that resonate more deeply with different patient populations may further improve the effectiveness of the gamified approach. Additionally, other gamification strategies, such as reward systems or adaptive challenges, could be investigated to assess their potential impact on patient outcomes.

A key next step is to conduct a larger-scale study with a control group to better assess the effectiveness of the gamified approach compared to traditional methods. This would allow for a more robust statistical analysis and provide stronger evidence of the intervention’s benefits in improving medication adherence and patient awareness. Expanding the study to diverse patient demographics would also offer insights into the approach’s generalizability and scalability.

### Conclusion

Smart-Meds is a promising app; although one-third of the participants dropped out immediately, the remaining participants used the app regularly. The satisfaction of users was high, and participants would recommend the app to others. Our results show an improvement in the self-reported medication adherence scale after 4 weeks of app use. Although gamification has been successful in boosting behavior change in several contexts, it seems to have a limited impact on our specific population. Therefore, additional research should be conducted with the end user to design a story that boosts their motivation. On the experimental side, a larger study with a controlled design like a randomized controlled trial is needed to confirm our results.

## Abbreviation

ACS: acute coronary syndrome
BMQ: Beliefs About Medication Questionnaire
CR: cardiac rehabilitation
SEAMS: Self-Efficacy for Appropriate Medication Use Scale
SUS: System Usability Scale
